# Supplementary material for: In situ X-ray imaging of defect and molten pool dynamics in laser additive manufacturing
Source: Nat Commun. 2018 Apr 10;9:1355. doi: 10.1038/s41467-018-03734-7 (PMC5893568; doi:10.1038/s41467-018-03734-7)
Supplement: Supplementary file 1 — Supplementary Information [file 41467_2018_3734_MOESM1_ESM.pdf]

In situ X-ray imaging of defect and molten pool dynamics in laser additive manufacturing

Chu Lun Alex Leung[1*], Sebastian Marussi[1], Robert C. Atwood[2], Michael Towrie[3], Philip J. Withers[1], Peter D. Lee[1*]

[1] School of Materials, The University of Manchester, Oxford Rd, Manchester, M13 9PL, UK

[2] Diamond Light Source Ltd, Diamond House, Harwell Science & Innovation Campus, Didcot, Oxfordshire, OX11 0DE, UK

[3] Central Laser Facility, Research Complex at Harwell, Science & Technology Facilities Council, Rutherford Appleton Laboratory, Didcot, Oxfordshire OX11 0QX, UK

*emails: peter.lee@manchester.ac.uk and alex.cl.leung@manchester.ac.uk

1. Supplementary Methods

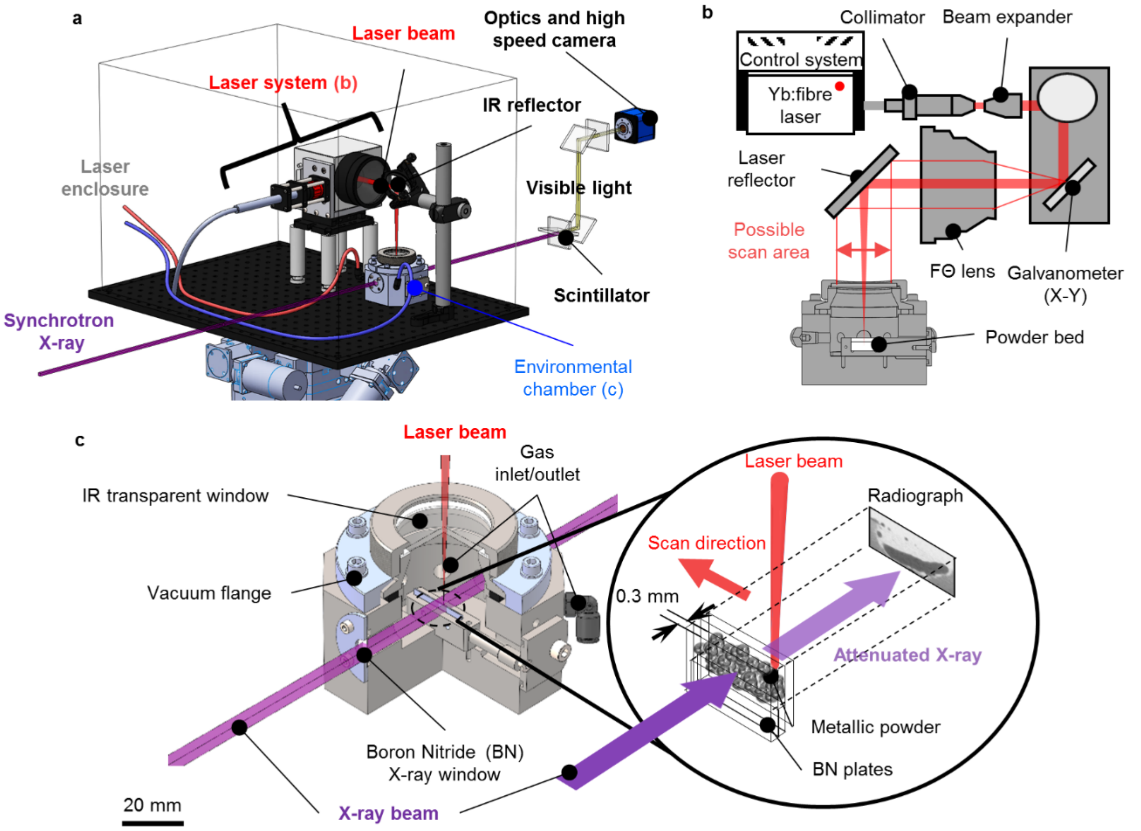

Supplementary Figure 1: Experimental setup for the *in situ* laser additive manufacturing process replicator (LAMPR). a, LAMPR consists of an environmental chamber, a laser system, an IR reflector and a laser enclosure. The X-ray beam illuminates the environmental chamber via the X-ray window with the laser beam travelling from right to left (red arrow). **b,** A detailed schematic of the laser system demonstrating how the laser beam is collimated, expanded and focused before it reaches the powder bed; the laser beam can be steered in the horizontal plane by an X-Y galvanometer at various scan speeds. **(c)** Sectional views of the environmental chamber with an inset to demonstrate how the X-ray interacts with the metallic powder, which is held inside a cavity, sandwiched between X-ray translucent boron nitride (BN) plates.

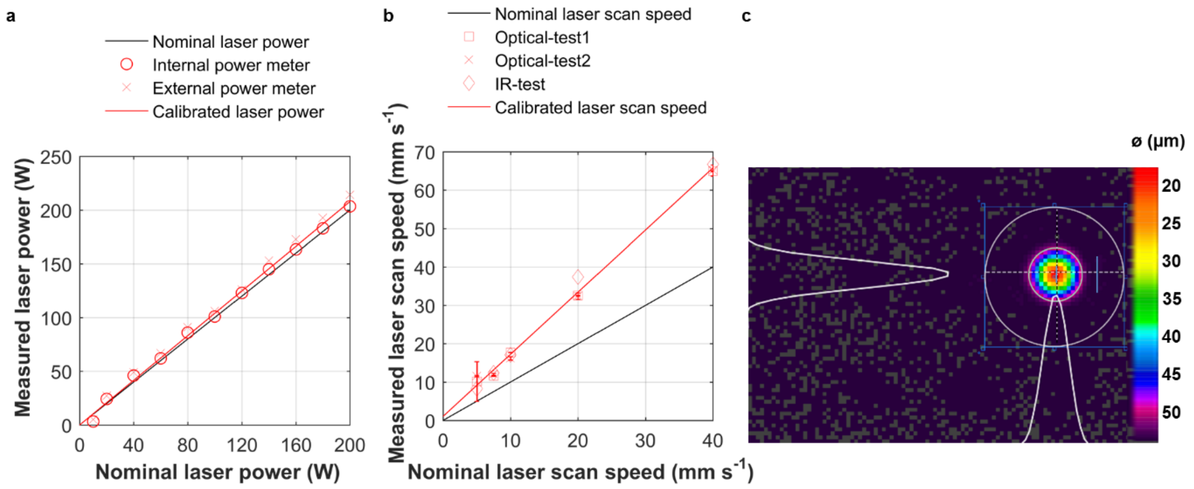

Supplementary Figure 2: Laser beam characteristics. a, Measured laser power versus nominal laser power. **b,** Measured laser scan speed versus nominal laser scan speed. **c,** A laser beam profile fitted by a Gaussian fitting, see colour map for the beam diameter. The full laser characteristics are given in **Table 1**.

The laser power and scan speed of the LAMPR were measured after the experiments. The results are very consistent but differ from the nominal values as shown in **Supplementary Figure 2a-b**. Hence, we used the measured values in the main text.

The laser power was measured by two different methods as shown in **Supplementary Figure 2a**: (1) an internal power meter of the laser system and the official R4 Laser GUI (SPI Lasers Ltd, UK), and (2) an external power meter, FL250A-SH-V1 with a Nova display (Ophir Spiricon Europe GmbH, Germany). The black line represents the nominal laser power whereas the red line represents the calibrated laser power, which is a linear fit of the measured laser power. For the nominal laser power of 100, 150 and 200 W, they are calibrated as 106, 157 and 209 W, respectively.

To calibrate the path length of the laser beam, we used an embedded visible light laser beam (λ = 630 nm) inside the SPI laser system to scan multiple times on an alignment screen with a 10 mm target grid (Thorlabs Inc., USA) while adjusting the X-Y scaling factor of the laser scanning system using its control software, Digistrut (Laser control system Ltd, UK). After we calibrated the path length of the laser beam, we recorded the duration of each scan process using two optical imaging methods for determining the laser scan speed.

For the optical tests, we scanned two lines, a 4 mm (Optical-test1) and a 40 mm (Optical-test2), using an embedded visible light laser beam inside the SPI laser system, and simultaneously recorded the scan process at 240 fps by an iPhone6 (Apple Inc., USA). This indicated that the true laser scan speed of the tested range was higher than the nominal value. To confirm this, we scanned a 4 mm line using the 1070 nm laser beam while recording the scan process using a high speed camera at 1000 fps, Phantom V7.3 (Vision Research Inc., USA) with an NIR illumination source (λ = 960 nm) and a notch filter (λ =1000 nm ± 50 nm). The results matched well with the iPhone measurements. The black line represents the nominal laser scan speed whereas the red line represents the calibrated laser scan speed, which is a linear fit of the measured laser scan speed. For 5, 7.5, 10, 20 and 40 mm s^{-1}, the calibrated scan speeds are 9, 13, 17, 34 and 68 mm s^{-1}, respectively.

The laser spot size was recorded using a CCD camera with a pixel size of 3.69 μm through a unique wedge system (which consists of two quartz wedges and two NIR wedges) (Ophir Spiricon Europe GmbH, Germany). This wedge system reduced the intensity of the 1070 nm laser beam by 0.00016% to prevent the laser beam damaging the CCD camera. The beam profile was displayed using the BeamGauge software (Ophir Spiricon Europe GmbH, Germany) and the spot size was estimated by fitting a Gaussian function as shown in **Supplementary Figure 2c.**

2. Supplementary Note 1

Supplementary Figure 3a shows a scanning electron image of the Invar 36 powder. We performed an elemental analysis using ANALYZER (Aztec, Oxford Instrument) in the same field of view. The elemental composition of the powder is shown in **Supplementary Figure 3b.** The oxygen concentration of 5.3 weight percent (wt %) indicates that the as-received powder has an oxidised surface.

We segmented the particles using Otsu's method[1], and then separated them by the watershed algorithm as implemented in the imaging toolbox of MATLAB 2016a (MathWorks, USA). The equivalent area-diameter of the segmented objects is shown in **Supplementary Figure 3c** and the particle size distribution is shown in **Supplementary Figure 3d**.

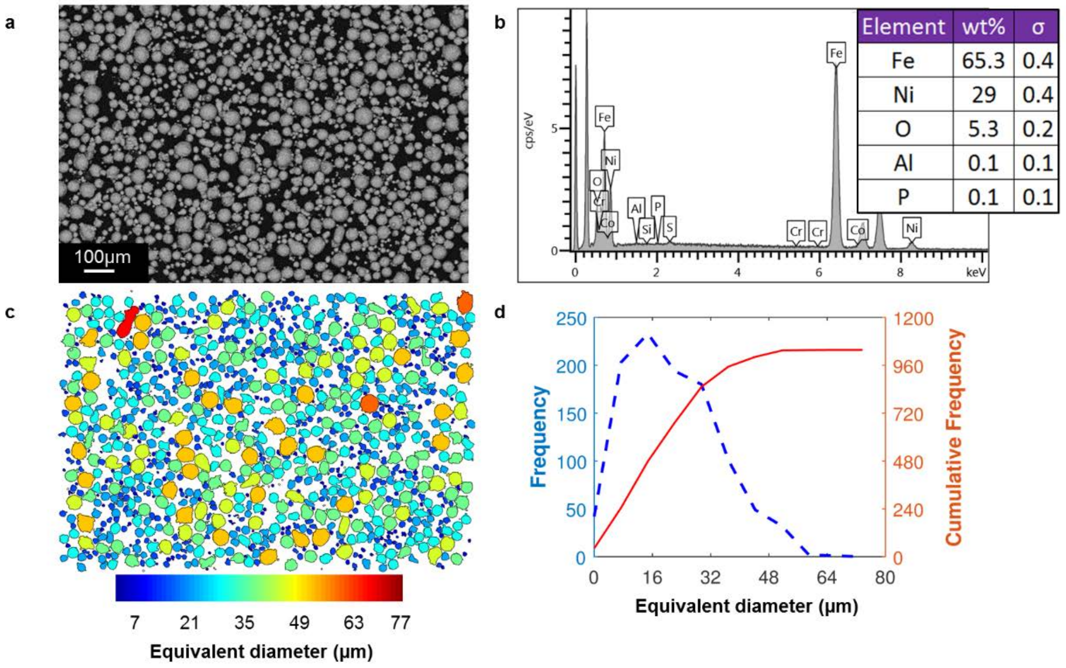

Supplementary Figure 3: Characterisation of Invar 36 powder: a, A backscatter scanning electron image of Invar 36 powder. **b,** An Energy-Dispersive X-ray (EDX) spectrum of (a) indicates the presence of oxidised particles. **c**, Powder particles in **(a)** are labelled with the area-equivalent diameter of individual particles. **d**, Particle size distribution of Invar 36.

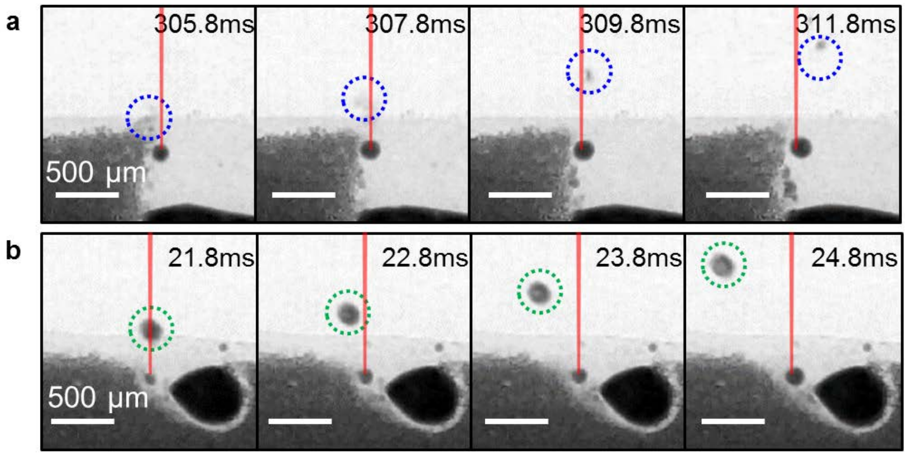

Supplementary Figure 4: Additional spatter mechanisms: a, Formation of droplet spatter by laser melting of powder spatter (see blue dotted circle) and **b,** laser-induced gas expansion inside a droplet spatter (see green dotted circle). Each cropped image is labelled with a red laser beam. Scale bars, 500 μm.

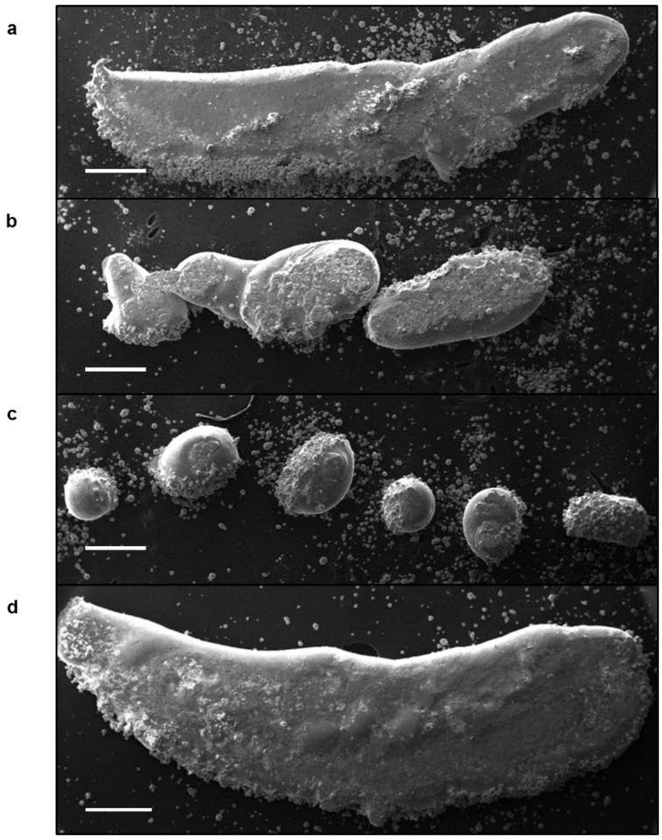

Supplementary Figure 5: Selected secondary electron images illustrate the final morphologies of the melt features. a, Example of a continuous track (*P* = 157 W, *v* = 9 mm s^{-1}, *LED* = 17.4 J mm^{-1}), **b,** discontinuous track (*P* = 106 W, *v* = 34 mm s^{-1}, *LED* = 3.1 J mm^{-1}), **c,** Spheroidisation of metal beads induced by balling (*P* = 106 W, *v* = 68 mm s^{-1}, *LED* = 1.6 J mm^{-1}), **d,** the dual-layer track, *i.e.* MT2 (*P* = 209 W, *v* = 13 mm s^{-1}, *LED* = 16.1 J mm^{-1}). Scale bars, 500 µm.

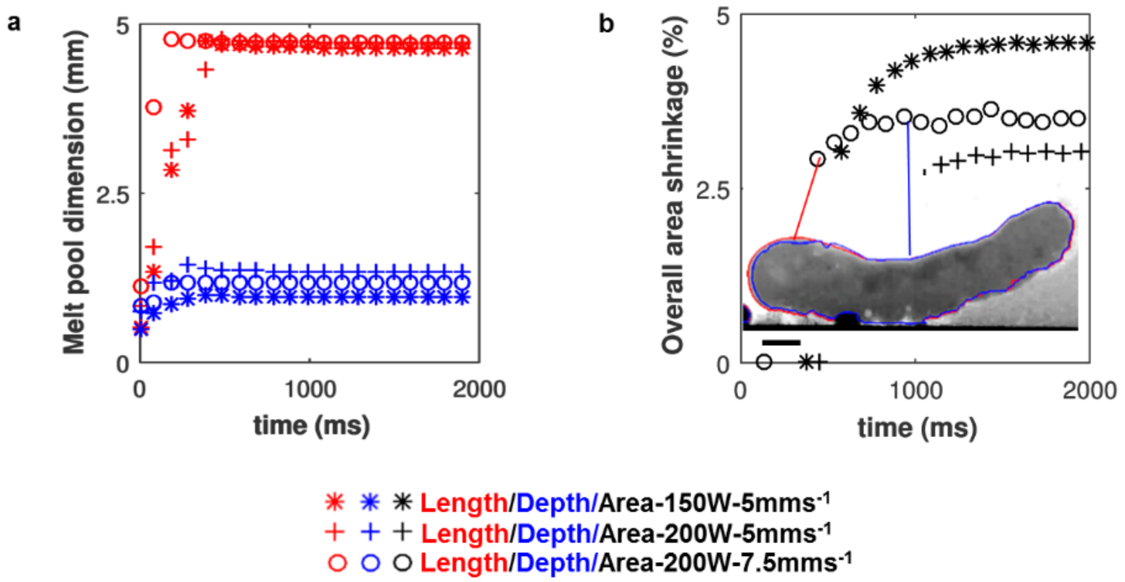

Supplementary Figure 6: Time-resolved melt track quantification of three continuous melt tracks (orange boxes) by image analysis: **a,** the length (red) and width (blue) of the molten pool as well as **b,** the overall area shrinkage of the molten pool (%) (black). The inset illustrates the shape of the melt track after the laser switches off (334ms), which overlays with contours line of the melt track at 334 ms (red) and 998 ms (blue). Scale bar, 500 μm.

3. Supplementary Tables

Supplementary Table 1: Extracted quantification results from Supplementary Figure 5

P (W)	*v* (mm s^{-1})	LED (J mm^{-1})	Length (mm)	Nominal length (mm)	Deviation in length (%)	Maximum shrinkage (%)
157	9	17.4	4.76	4	18.9	4.59
209	9	23.2	4.77	4	19.3	3.04
209	13	16.1	4.78	4	19.5	3.64

Supplementary Table 2: Laser beam characteristics of the SPI R4 laser system

Parameter	Units	Specification	Measured
Central emission wavelength	nm	1070 ± 10	Pass
Emission bandwidth	nm	< 4.0	Pass
Modulation rise time	µs	< 20	Pass
Modulation fall time	µs	< 10	Pass
Beam diameter (Incoming)	mm	5.0 ± 0.7	5.3
Beam divergence	mrad	≤ 0.45	0.27
M^2	-	≤ 1.10	1.03
Circularity (%)	%		97.1
Eccentricity	%	≤ ± 1.2	Pass
Concentricity	mm	≤ ± 2.0	Pass
D4σX	µm	49.5	50.9
D4σY	µm	49.5	49.8

4. Supplementary References

1. Otsu, N. A Threshold Selection Method from Gray-Level Histograms. *IEEE Trans. Syst. Man. Cybern.* **9,** 62–66 (1979).
