# Supplementary material for: In situ X-ray imaging of defect and molten pool dynamics in laser additive manufacturing
Source: Nat Commun. 2018 Apr 10;9:1355. doi: 10.1038/s41467-018-03734-7 (PMC5893568; doi:10.1038/s41467-018-03734-7)
Supplement: Supplementary file 3 — Description of Additional Supplementary Files [file 41467_2018_3734_MOESM3_ESM.docx]

**Description of Additional Supplementary Files**

File Name: Supplementary Movie 1

Description: Time-lapse video showing the formation of a single layer melt track (MT1) by laser melting of Invar 36 powder at a laser power of 209 W and a laser scan speed of 13 mm s^-1^.

File Name: Supplementary Movie 2

Description: Time-lapse video showing the formation of discontinuous tracks by laser melting of Invar 36 powder at a laser power of 106 W and a laser scan speed of 34 mm s^-1^.

File Name: Supplementary Movie 3

Description: Time-lapse video showing the formation of balling by laser melting of Invar 36 powder at a laser power of 106 W and a laser scan speed of 68 mm s^-1^.

File Name: Supplementary Movie 4

Description: Illustration of melt features being tracked during laser melting of Invar 36 at a laser power of 157 W and a laser scan speed of 9 mm s^-1^. The red boundary highlights the segmented molten pools or melt track that overlaid to the denoised radiographs.

File Name: Supplementary Movie 5

Description: Time-series images showing the formation of a second layer melt track (MT2) by laser melting of Invar 36 powder at a laser power of 209 W and a laser scan speed of 13 mm s^-1^.

File Name: Supplementary Movie 6

Description: A droplet spatter is tracked from the onset of the laser melting of Invar 36 powder at a laser power of 209 W and a laser scan speed of 13 mm s^-1^. The spatter trajectory is shown by the red line.

File Name: Supplementary Movie 7

Description: After laser melting of Invar 36 powder at a laser power of 209 W and a laser scan speed of 13 mm s^-1^ , the pore motions are tracked during the cooling stage. Each colour dot highlights the centre of individual pores. The flow pattern emphasises that the pores follow the centrifugal Marangoni convection during laser melting.
